# Supplementary material for: What factors have influenced quality of life in people with dementia and their family carers during the COVID-19 pandemic: a qualitative study
Source: BMJ Open. 2022 Feb 10;12(2):e053563. doi: 10.1136/bmjopen-2021-053563 (PMC8845096; doi:10.1136/bmjopen-2021-053563)
Supplement: Supplementary data [file bmjopen-2021-053563supp001.pdf]

TfD C19 Study

Topic Guide/Interview schedule

Interview by phone or video conferencing

Introduction

My name is …... I am a researcher on the COVID-19 study

Remind participant about study – to understand the impact of C19 on the quality of life of people living with dementia.

Aim for today:

- *to complete an interview / have an informal chat about your experiences of COVID-19*

If you are happy to go ahead

- *is it convenient to do it today?*
- *Audio recording*
- *Anonymized and confidential*
- *40-50 minutes*
- *Keep to time*
- *Can stop / take a break at any time*

Do you have any questions?

Are you happy to go ahead?

To do: Test dictaphone

Record carer ID, date, start time

Introduction

I am going to start by asking you a question about lift before the pandemic and then ask you to tell me more about the general impact of the C19 restrictions on you and the person you care for (use their name/relationship) . I will then ask you about their (person with dementia) experiences in more depth and then finish the interview with exploring your experiences in more detail.

1) ***Can you tell me a little bit about the life before the pandemic for you and for the person who you care for (name)***

2) ***Can you tell me impact of the C19 restrictions on your day to day life?***

3) ***Can you tell me more about the general impact of C19 restrictions on day to day life on the person (name) who you care for?***

4) ***In more detail, what has been the impact on the (name) person with dementia?***

- *(Prompts) Positive/Negative? What has been most impactful?*
- *What has the person with dementia understood about what has been happening? Especially if there has been no/reduced contact with carer?*
- *What has been the impact of reduced social interaction with others? What about the loss of physical contact*
- *Has there been any impact on their dementia? If so, in what way? Why is this?*
- *What has been the impact on their routine? What has been the impact on their physical health (including mobility)?*
- *Has there been any impact on their general well-being and mental health – how would you say that they are feeling?*
- *How they found any support or solutions which have been particularly helpful/useful?*

5) ***Impact of the carer***

- *What has been the impact on you? (Positive/Negative)*

- *Prompts (to explore) What impact has there been on:*
- *Your ability to meet your own personal needs*
- *Your well-being,*
- *Your relationship with the person with dementia – (If you are not living with/seeing the person with dementia – how has this been for you? What might be the impact on your relationship?)*
- *Your feelings about the future*
- *how supported do you feel?*
- *What impact is there on your social networks/isolation?*
- *How has this impacted upon your sense of control?*
- *How has the impact on the person with dementia affected you?*
- *Has the person with dementia received a DNR (do not resuscitate) notification – if yes, what is the impact of this on you?*

- *Any other areas which they feel have been impacted upon?*

TfD C19 study carer topic guide

- *How have you found any support or solutions (national local) which have been particularly helpful/useful?*

6) Other?

Anything which we haven't discussed which might be relevant?

Is there anything from the pandemic experience which you would like to 'keep'

7) Debrief

- *How did you find the interview/how are you feeling now?*
- *Would you be interested in doing another interview later in the year should we be able to do some further follow?*
- *Re-iterate that he/she can call researcher if he/she feels distressed after call*
- *Re-iterate that we will do a follow up call if he/she has been distressed*
- *What is he/she going to do next?*

TfD C19 study carer topic guide
