# Supplementary material for: What factors have influenced quality of life in people with dementia and their family carers during the COVID-19 pandemic: a qualitative study
Source: BMJ Open. 2022 Feb 10;12(2):e053563. doi: 10.1136/bmjopen-2021-053563 (PMC8845096; doi:10.1136/bmjopen-2021-053563)
Supplement: Author's manuscript [file bmjopen-2021-053563.draft_revisions.pdf]

BMJ Open

BMJ Open is committed to open peer review. As part of this commitment we make the peer review history of every article we publish publicly available.

When an article is published we post the peer reviewers' comments and the authors' responses online. We also post the versions of the paper that were used during peer review. These are the versions that the peer review comments apply to.

The versions of the paper that follow are the versions that were submitted during the peer review process. They are not the versions of record or the final published versions. They should not be cited or distributed as the published version of this manuscript.

BMJ Open is an open access journal and the full, final, typeset and author-corrected version of record of the manuscript is available on our site with no access controls, subscription charges or pay-per-view fees (http://bmjopen.bmj.com).

If you have any questions on BMJ Open's open peer review process please email info.bmjopen@bmj.com

BMJ Open

What factors have influenced quality of life in people with dementia and their family carers during the COVID-19 pandemic: a qualitative study

Journal:	*BMJ Open*
Manuscript ID	bmjopen-2021-053563
Article Type:	Original research
Date Submitted by the Author:	20-May-2021
Complete List of Authors:	Daley, Stephanie; Brighton and Sussex Medical School, Centre for Dementia Studies; Brighton and Sussex Medical School<br>Akarsu, Nazire; Sussex Partnership NHS Foundation Trust<br>Armsby , Elise; Sussex Partnership NHS Foundation Trust<br>Farina, Nicolas; Brighton and Sussex Medical School, Centre for Dementia Studies<br>Feeney, Yvonne; Brighton and Sussex Medical School, Centre for Dementia Studies<br>Fine , Bethany; Sussex Partnership NHS Foundation Trust<br>Hughes, Laura; Brighton and Sussex Medical School,<br>Pooley, Joanna; Sussex Partnership NHS Foundation Trust<br>Tabet, Naji; Brighton and Sussex Medical School<br>Towson , Georgia; Sussex Partnership NHS Foundation Trust<br>Banerjee, Sube; University of Plymouth Faculty of Health and Human Sciences, Centre for Dementia Studies
Keywords:	Dementia < NEUROLOGY, Old age psychiatry < PSYCHIATRY, COVID-19

SCHOLARONE™
Manuscripts

BMJ

I, the Submitting Author has the right to grant and does grant on behalf of all authors of the Work (as defined in the below author licence), an exclusive licence and/or a non-exclusive licence for contributions from authors who are: i) UK Crown employees; ii) where BMJ has agreed a CC-BY licence shall apply, and/or iii) in accordance with the terms applicable for US Federal Government officers or employees acting as part of their official duties; on a worldwide, perpetual, irrevocable, royalty-free basis to BMJ Publishing Group Ltd ("BMJ") its licensees and where the relevant Journal is co-owned by BMJ to the co-owners of the Journal, to publish the Work in this journal and any other BMJ products and to exploit all rights, as set out in our licence.

The Submitting Author accepts and understands that any supply made under these terms is made by BMJ to the Submitting Author unless you are acting as an employee on behalf of your employer or a postgraduate student of an affiliated institution which is paying any applicable article publishing charge ("APC") for Open Access articles. Where the Submitting Author wishes to make the Work available on an Open Access basis (and intends to pay the relevant APC), the terms of reuse of such Open Access shall be governed by a Creative Commons licence – details of these licences and which Creative Commons licence will apply to this Work are set out in our licence referred to above.

Other than as permitted in any relevant BMJ Author's Self Archiving Policies, I confirm this Work has not been accepted for publication elsewhere, is not being considered for publication elsewhere and does not duplicate material already published. I confirm all authors consent to publication of this Work and authorise the granting of this licence.

For peer review only

What factors have influenced quality of life in people with dementia and their family carers during the COVID-19 pandemic: a qualitative study

Authors:

Dr Stephanie Daley[a]

Nazire Akarsu[a]

Elise Armsby[b]

Dr Nicolas Farina[a]

Yvonne Feeney[a]

Bethany Fine[b]

Dr Laura Hughes[a]

Joanna Pooley[a]

Dr Naji Tabet[a]

Georgia Towson[a]

Professor Sube Banerjee[c]

Affiliations and addresses:

a. Centre for Dementia Studies, Brighton and Sussex Medical School, UK
b. Dementia Research Unit, Sussex Partnership NHS Foundation Trust, UK
c. Faculty of Health, University of Plymouth, UK

Corresponding Author

Dr Stephanie Daley

Address: Brighton & Sussex Medical School, Centre for Dementia Studies, Trafford Building, Room 101, University of Sussex, Falmer, BN1 9RY.

Email: s.daley@bsms.ac.uk

Tel: 00 447393 761947

For peer review only

Abstract

Objectives: The COVID-19 pandemic has led to significant disruption to health and social care services. For people with dementia and their family carers this is problematic, as a group who rely upon timely and responsive services to live well with the condition. This study has sought to understand how COVID-19 has affected the quality of life of people diagnosed with dementia and their family carers

Design: Our mixed-methods study was nested in a larger cohort study of an education programme, Time for Dementia

Setting: The study took place in the South-East of England.

Participants: Existing study participants, family carers were approached about the COVID-19 nested study. A purposeful sample of participants were invited to take part in in-depth qualitative interview. The sample included family carers in a range of different caring situations.

Measurement: Interviews were undertaken remotely by telephone. Interviews sought to understand quality of life before the pandemic, impact of the restrictions on both the person with dementia and family carer, role of services and other agencies as well as supportive factors. Data were analysed using thematic analysis.

Results: 16 family carers were interviewed. Seven themes were identified from our analysis 1) decreased social interaction, 2) reduced support, 3) deteriorating cognitive & physical health for the person with dementia, 4) decreased carer well-being 5) difficulties understanding COVID-19 restrictions, 6) limited impact for some, and 7) trust and relationship with care home. There was little change between themes during the first and second wave of national lockdowns.

Conclusions: Our study provides an understanding the short-term impact of COVID-19 on the quality of life of people with dementia and their family carers. Our findings suggest that recovery between the first and second wave of the restrictions did not automatically take place.

For peer review only

Strengths and limitations of this study

- Strengths of this study are the inclusion of a range of family carers across different caring situations, as well as data collection in ‘real time’.
- A further strength is that the study did not have an *a priori* hypothesis, and as such took an inductive ‘bottom up’ approach to data collection and analysis, with strong lived experience involvement.
- Limitations include the use of proxy reports from family carers about the impact of the pandemic on the QoL of people with dementia, rather than self-report
- The study sample lacks ethnic diversity as well as those with a recent diagnosis of dementia.

For peer review only

Introduction

The COVID-19 pandemic and subsequent local, regional, and national lockdowns have led to significant disruption to health and social care services. For people with dementia and their family carers this is problematic. There are currently 850,000 people with dementia living in the UK (1) who rely upon timely and responsive services to live well with the condition. The abrupt closure and reduction of many health and community support services arising from the pandemic is of concern for this vulnerable group. The UK government introduced a range of restrictions (shielding for the most medically vulnerable, self-isolation, and social distancing) to help reduce the spread of COVID-19, and to protect older and vulnerable people. Commentaries exist about how the these restrictions may be affecting people with dementia and carers (2) however the full impact of these restrictions has yet to be assessed.

Emerging anecdotal reports highlight that along with the disruption to formal dementia services, restrictions have led to a further loss of support from family and friends for people with dementia and family carers (3). Since the first national lockdown in March 2020 in the UK, there has been an increased number of people telephoning the support services provided by the Alzheimer's Society due to concerns about increased cognitive and functional deterioration in family members with dementia (4). In addition, feedback from webinars (led by the advocacy group; 3 Nations Dementia Working Group) revealed that people with dementia had significant concern about the withdrawal of services leading to a sense of abandonment, along with anxiety about the potential for a permanent loss of services (5).

However, empirical data to address these concerns is sparse. One qualitative study which looked at the impact of reduced social support with 42 carers and 8 people with dementia identified that that the sudden reduction in services had significantly affected both family carers and people with dementia (6). This research identified three key themes: loss of control, uncertainty (about current and future provision), and a need to adapt to 'the new normal' (including remote rather than face to face contact). A survey of 191 family carers of people with dementia found an 83% increase of caring tasks as a consequence of COVID-19, with over 50% reporting that they felt unable to fulfil the caring role adequately (7).

The emerging literature highlights the substantial impact of the loss of both formal and voluntary sector services, a fear of services not resuming, increased carer burden, and concern about increased cognitive decline in the person with dementia. We are not aware of any research undertaken during this period which has specifically sought to assess the impact of the COVID-19 pandemic upon the Quality of Life (QoL) of either people with

For peer review only

dementia or family carers. We know that living with a diagnosis of dementia, or caring for somebody with dementia is impactful upon all of the factors known to affect QoL, including physical health, psychological state, independence, social relationships, personal beliefs and environmental supports (8). Whilst factors influencing QoL in both people with dementia and their carers differ, their outcomes are inextricably linked; with carers being a vital determinant of QoL and positive outcomes for people with dementia, and vice versa. Therefore, in this study we aimed to understand how COVID-19 has affected the QoL of people diagnosed with dementia and their family carers

Methods

Study design

Our study was nested in a larger cohort study, Time for Dementia (9). Time for Dementia is an undergraduate education programme in the South-East of England, whereby people with dementia and their carer teach healthcare students about living with the condition. During the pandemic period, there has been remote contact (video conferencing or telephone) between carers, the person with dementia and their family carer on a termly basis.

Sample and setting

We recruited family carers of people with a diagnosis of dementia from the Time for Dementia study. The study definition of family carers included married and unmarried partners, children, siblings, extended family and close friends. The term ‘family carers’ is since it was preferred by our lived experience advisory group. We sampled for variation in characteristics of family carers, using the socio-demographic information provided by participants. From discussion with our lived experience advisory group, we agreed that these characteristics should include: resident and non-resident family carers, spousal and non-spousal family carers, those who were shielding, and family carers of people with dementia in care homes. Due to the difficulty in assessing capacity over the telephone, people with dementia were not interviewed as part of this study. The study was approved by the NHS Health Research Authority London Queen Square Research Ethics Committee (15/LO/0046).

Procedure

Participants in the Time for Dementia evaluation were approached by a research worker about the COVID-19 nested study. Those who were interested were sent a study information sheet, and verbal consent was obtained by those who wished to take part. Quantitative

For peer review only

measures of QoL were undertaken, along with socio-demographic information and are reported separately. A purposeful sample of participants were asked about undertaking an in-depth qualitative interview. The sample included family carers in a range of different caring situations.

Topic guides were developed from an initial review of the literature, and key topic areas included: life before the pandemic, impact of the restrictions on both the person with dementia and family carer, role of services and other agencies and supportive factors. Additionally, the topic guide for interviews were amended to explore change over the pandemic period, and also for family carers of people living in a care home.

Interviews were carried out between May and November 2020 by JP, SD and NA. Twelve interviews were undertaken in the first lockdown (May and June 2020), and ten interviews were undertaken during the second lockdown (November 2020). Six participants were interviewed twice, and family carers of people living with dementia living in a care home (n=4) were interviewed once during the second lockdown. Interviews were stopped when data reached thematic saturation. Interviews were undertaken by telephone and lasted between 30 and 60 minutes

All interviews were audio-recorded, transcribed verbatim and checked for accuracy.

Analysis

Data analysis commenced in July 2020 using thematic analysis. The process started with descriptive coding of two transcripts by two researchers (SD and JP) who manually coded the transcripts by giving descriptive codes to meaningful segments of text. The researchers met to review their respective preliminary codes and identify areas of differences and to develop an initial framework. The remaining 10 transcripts (from the first round of interviews) were coded to develop a focussed framework. The topic guide was amended at this stage, to gain further understanding of developing themes and to explore change over time. During the second phase of the analysis (second round of interviews), 6 transcripts were coded by SD and JP. GT and SD undertook a separate analysis of the care home transcripts, using the existing study coding framework, generating additional codes and themes as necessary.

The computer software package, NVivo 12 (QSR International, 2018) was used to enable the systematic collation and review of the data grouped within each code. The researchers met regularly to compare coding and data between existing and new transcripts in order to check on the use of codes for consistency and areas of uncertainty. The researchers also jointly reviewed data collated within each code, to identify relationships between codes, as

For peer review only

well as areas of uncertainty, coming to an agreement once discussed. In the last phase of the analysis, the care home data was incorporated, and a final framework was produced from which seven over-arching themes were identified.

Rigour in the research process was supported in two ways. First, the lead researcher (SD) met frequently with the researchers for academic supervision to review coding, the development of themes and reflexivity. Second, all researchers kept fieldwork diaries.

Patient and public involvement

People with dementia and their family carers were involved throughout the research process though a specific Lived Experience Advisory Group (LEAP) which was established for the study. The group reviewed the study design, participant-facing documentation, the topic guide on two occasions as well as emerging and final themes. The group have also been involved in developing dissemination material for participants, as well as for local services and stakeholders.

Results

The 16 participants were predominantly White British/European (94%), spousal carers (64%). Half of participants were co-resident with the person with dementia (50%). Table

Table 1 shows the participant characteristics.

Insert Table 1 here

Themes

Our analysis identified seven themes: 1) decreased social interaction, 2) reduced support, 3) deteriorating cognitive & physical health, 4) decreased carer well-being 5) difficulties understanding COVID-19 restrictions, 6) limited impact for some, and 7) trust and relationship with care home. Themes are illustrated in more depth in Table 2.

Insert Table 2 here

These themes were all identified in relation to perceived impact of COVID-19 on the QoL of the person with dementia and family carer. For the majority of themes, there was little change between the first and second lockdown. With the exception of family carers of people with dementia living in care homes, those interviewed highlighted day-to-day life before the pandemic as including opportunities for social interaction and for support for both the person with dementia and family carer with benefits to each. Many of the themes

For peer review only

identified are inter-related both between themselves and between the person with dementia and their carer, as shown in Figure 1.

Insert Figure 1 here

Theme 1: Decreased social interaction

All family carers reported that the significant reduction in face to face social interaction and contact with others impacted upon QoL for themselves and the person with dementia. Feelings of loneliness and isolation were frequently reported by family carers, especially for those who would have ordinarily relied upon social contact as a source of respite from their caring role. Lack of social interaction led to sense of reduced informal support (from others) and was impactful across the majority of other themes identified in the analysis.

Family carers expressed a sense of time being short for the person with dementia, and precious time with family members being lost. Concern about the potential risks of catching the virus appeared to result in isolation for many family carers who reported that they had avoided social opportunities, declined support from services both for themselves and the person with dementia.

Family carers of those in care homes were all spousal carers, and all reported that being unable to see the person with dementia was very impactful on their QoL, leading to significant feelings of loneliness.

Theme 2: Reduced support

Family carers stated that pre-COVID informal face-to-face support provided by friends and family was highly valued, and positively impactful on their own QoL as well as the QoL of the person with dementia. Since the COVID-19 pandemic, this type of support had reduced for many, primarily due to social distancing regulations, including shielding. In the absence of face-to-face support, family carers recognised the benefit of remote contact with friends and family. An increased reliance on technology (such as phone and video calls) was seen as being problematic for both the family and for the person with dementia. Family carers reported that it is more difficult to offload fully about current caring challenges by phone or video calls, either because the person with dementia was present or because there was less available time. For those with dementia who were unfamiliar with technology such as video calls, family carers reported that using them was often confusing, and of limited value.

A sharp reduction of services such as day care, respite, community groups and non-urgent healthcare was widely reported, accompanied by concerns about the future resumption of

For peer review only

support services. For a small number of those interviewed, formal respite services had continued or had resumed, and this was highly valued.

Family carers also reported that some professional support groups adapted to provide some services remotely, and whilst this was valued, there was dissatisfaction with the depth of connection and support possible through remote contact.

Due to COVID-19, the process of obtaining a care home place had become more difficult, with three family carers reporting that the transition into the care home had ultimately been made following an acute hospital admission.

Theme 3: Deteriorating cognitive and physical health

Many of the family carers interviewed expressed concern about increased cognitive decline in the person with dementia, linking this to a lack of stimulation, from reduced social interaction with others (family, friends and dementia support services). Several of carers interviewed noted that the decline in cognition could also have been attributable due to the natural progression of the condition.

Where the person with dementia was community dwelling, family carers reported that reduced levels of social and support activity disrupted the routine of the person with dementia, negatively impacting upon their motivation and fatigue levels.

Family carers reported worsening physical health in the person with dementia for those who were community dwelling. Reduced mobility was highlighted as a particular concern, linked to the wider lack of activity, along with an increased rate of joint pain, and for some, an increase in urinary tract infections.

Theme 4: Decreased carer well-being

For most family carers, the pandemic had led to increased caring tasks arising from increased dependence of the person with dementia and a reduced level (service or family/friend) of support. Increased caring tasks, along with the lack of respite opportunities for a break had led to a sense of burnout for some. Feelings of frustration, anger and resentment about the situation from some of those interviewed was expressed, increasing over time between the first and second lockdown.

Theme 4: Decreased carer well-being

For peer review only

For most family carers, the pandemic had led to increased caring tasks arising from increased dependence of the person with dementia and a reduced level (service or family/friend) support. Increasing caring, along with the lack of respite opportunities for a break had led to a sense of burnout for some. Feelings of frustration, anger and resentment from some of those interviewed was expressed, increasing over time between the first and second lockdown.

Additionally, for family carers, increased caring tasks and the need for direct supervision of the person with dementia impacted upon their ability to meet their own needs. The majority of family carers interviewed described a loss of freedom in being able to undertake the activities which they wanted to undertake, and a sense of a loss of control and certainty. Some of those interviewed described feeling suffocated, lonely and frustrated.

Theme 5: Difficulties understanding COVID-19 restrictions

Difficulties in understanding the pandemic and its related restrictions was reported as being problematic for people with dementia. Carers reported that for some of those with dementia, the restrictions were often forgotten, or that they were too confusing, or difficult to understand. For many family carers, they reported that they frequently needed to explain the pandemic and remind the person with dementia about restrictions (for example; need to avoid close contact with others) when outside the home. This also applied to people with dementia in care homes, as carers stated that they were constantly having to explain why they could not visit in person, or why they were not allowed to touch the person with dementia. This often led to the person with dementia becoming upset, or in some cases angry.

Theme 6: Limited impact from the pandemic

For a small number of people with dementia, carers reported that there was no perceived impact arising from the pandemic due their lack of understanding of the situation, and not remembering that they were not seeing family and friends less often.

Theme 7: Trust/relationship with care home

Where the person with dementia was in a care home, family carers identified the importance of maintaining a good relationship and trust with the care home as being pivotal to their QoL,

For peer review only

due to the family carer being unable to visit the home. Carers stated that care homes who facilitated good communication through phone calls, video calls or emails enabled a greater sense of ease and confidence. Having confidence in the care provided, and knowing that the person with dementia was safe and well looked after was perceived as essential for family carer QoL. One participant described poor care being delivered to the person with dementia, along with poor communication as having a negative impact on their QoL.

Maintaining contact between the carer and the person with dementia was achieved in a variety of ways, including the use of garden ‘pods’, or telephone contact. However, one participant reported that no alternative means of maintaining contact with the person with dementia was provided. The ability to maintain contact with the person with dementia impacted carer QoL, and increased contact with the person with dementia was associated with enhanced satisfaction with care and improved QoL for carers.

Discussion

We have identified seven themes arising from the COVID-19 pandemic which family carers reported to have affected QoL for people with dementia and family carers.

Reduced social interaction was the most impactful factor influencing QoL, and whilst its identification as a factor is not surprising, its mediating role on other factors is of note. For people with dementia; the value of social relationships on QoL in terms of social support, social integration and companionship (10) is well known. For family carers, the positive impact of social interaction from family and social networks on QoL is also well-established (11-13). However, in terms of the impact of the COVID-19 pandemic, the effect appears to be two-pronged, firstly it is sudden yet on-going sense of *loss of* social contact with meaningful others which is impactful, as well as actual impact of reduced contact with others. For the person with dementia, the reduced social contact was seen by the majority of family carers as increasing cognitive impairment in the person with dementia, reinforcing the importance of social contact as a protective factor against cognitive decline (14).

Reduced social interaction with others, and the perceived impact of this on the cognition of the person with dementia increased the burden on family carers, along with the loss of personal freedom and a decreased ability to meet their own needs and their overall well-being. Loss of personal freedom has been identified as a factor in the QoL of family members of people with dementia (11, 13, 15). Reduced family carer well-being is of concern, as if prolonged, this may impair the ability of family carers to look after the person

For peer review only

with dementia, and may lead to an increased rate of transition into care homes. Our findings indicate that the resumption of face-to-face social support services for people with dementia and their family carers should be prioritised. Furthermore a lessening of restrictions will bring with it opportunities for families and friends to socialise, which of highly valued for this group The impact of restriction in support on QoL is consistent with emerging research on the impact of COVID in relation to reduced formal support (6), and fears about services not resuming (5).

Our finding that the social restrictions from the pandemic have negatively impacted upon the physical health of the person with dementia has not yet been identified elsewhere, indicating that more research in this area is required. Interestingly, whilst worsening family carer physical health is factor known to influence the QoL of carers of people with dementia (16) this was not identified in this study, possibly because of the relatively short time period, or alternatively because it may have existed pre-COVID-19.

Within the context of a care home setting, the importance of trust and a good relationship between family carers and staff is clear. In our study, three of the four people with dementia in a care home had been transferred to the care home during the COVID-19 pandemic. Notwithstanding some of the wider challenges experienced by care homes in relation to COVID-19, the lack of face-to-face contact between family carers and staff can be seen as an additional obstacle to be overcome. The need for care homes to meaningfully involve family carers is care decisions is recognised as being significant in the development of both trust and a good relationship (17), and ordinarily trust in care staff evolves over time as a process (18), therefore, the absence of a 'normal' face-to-face relationship with family carers has been problematic. Further research into the experiences of care home staff and people with dementia living in care homes during the pandemic would be useful to understand these experiences more fully

Finally, whilst this study provides an understanding the short-term impact of COVID-19 on people with dementia and their family carers, we do not know what the longer-term impact might be. We also do not know how a return to 'normal' might be achieved, or what the 'new normal' might look like for people with dementia and their family carers, and what might help or hinder this process. It does appear that recovery from the impact of the first wave of restrictions did not automatically take place, and anecdotally we are aware that reductions in social support services has been prolonged, with many services still not having fully resumed. The provision of formal support during the pandemic to the study sample was highly variable, and there remain very few data about how dementia services responded to

For peer review only

the pandemic, and how they might adapt going forward to meet the post-pandemic challenges for this group.

There are three strengths to this study. First, we were able to include a wide range of family carers in order to understand the impact of COVID-19 across a range of caring situations. Second, interviews were undertaken during both national lockdown periods with data being collected in ‘real time’, without reliance on recall. Third, the study did not have an *a priori* hypothesis, and as such took an inductive ‘bottom up’ approach to data collection and analysis, supported by strong lived experience involvement. This has helped to strengthen the validity of the findings and kept the study alert to the current concerns of those living with dementia during this unprecedented period.

There are four main limitations to this study. First, we have taken proxy reports from family carers about the impact of the pandemic on the QoL of people with dementia. QoL is a subjective appraisal and it is well known that proxy reports assess QoL differently to self-appraisal by people with dementia (19). It is therefore possible that people with dementia may have identified other factors as being more or less impactful during the pandemic. Proxy-reports for the people with dementia living in care homes are also less likely to be accurate, as contact between family carers and the person with dementia may have been too limited for a more informed assessment of QoL. Second, there is a lack of ethnic diversity in the study sample. The limits the generalizability of these findings to the wide group of Black, Asian and other ethnic minority group members who have been disproportionately negatively affected by the pandemic overall, and for whom the impact for those living with dementia may be more substantial. Third, this study did not include family carers of somebody of a recent diagnosis of dementia. All of those interviewed had been caring for the person with dementia for a period of time and therefore, the factors identified were additional to their on-going experience of living with dementia and the degree to the pandemic impacted upon this. This study therefore does not specifically aid understanding to the impact of COVID-19 on the QoL of those with a recent diagnosis of dementia.

However, this study does provide a new and valuable information that contributes usefully to the emerging evidence base on the impact of the COVID-19 restrictions on people with dementia and their family carers. The finding here can help commissioners and service providers in developing and delivering the robust post-pandemic support that is needed by this vulnerable group.

For peer review only

Table 1: Participant characteristics

Characteristic	Mean (SD) (Range)	n	%
**Age**	65.63 (9.88) (48 – 83)	16	
**Gender**			
Female		9	56.3
**Ethnicity**			
White British/European		15	93.8
Asian/ Asian British		1	6.3
**Marital status**			
Married		10	62.5
Cohabiting		2	12.5
Separated / Divorced		2	12.5
Never married/ Single		2	12.5
**Relationship to person with dementia**			
Spouse/partner		10	62.5
Son/daughter		6	37.5
**Currently employed**			
Yes		4	25.0
**Current/ last occupation**			
Managers/directors/officials		3	18.8
Professional		3	18.8
Associate professional/ technical		2	12.5
Administrative/ secretarial		5	31.3
Skilled trades		2	12.5
Caring/leisure/service		1	6.3
**Geographical location**			
Urban		1	6.3
Rural		7	43.8
Suburbs		8	50.0
**Person with dementia**			
**Type of dementia**			
Alzheimer's disease		4	25.0
Vascular dementia		3	18.8
Early onset Alzheimer's disease		2	12.5
Mixed		7	43.8
**Residence**			
Co-resident with carer		8	50.0
Non-resident		8	50.0
**Person with dementia**			
Community dwelling		12	75.0
Care home resident		4	25.0
**Time since diagnosis**	3.4 years (2.20) (1.1 - 7.5)		
Less than 1 year		0	0.0
Between 1 and 3 years		9	56.3
Between 3 and 5 years		2	12.5
Over 5 years		5	31.3

For peer review only

Table 2 Summary of themes

Themes/Quotes
**Theme 1: Decreased Social interaction:** <br>*'very very isolating really, both for me and mum really erm, coz she misses, she obviously misses seeing everyone'* ***Family carer 12, resident son, (baseline)*** <br>*'um well I miss it, miss it terribly because I'm quite gregarious and I do miss it, probably y'know somebody, not having people to kind of talk to and have a laugh about things and um just generally just to offload really, um y'know that makes a difference'* ***Family carer 2, resident spouse, female (follow-up)*** <br>*'the other main one really was has not being able to see his grandchildren. And because it's hard for anybody to know you know and I've sat with him while he's cried and gone "I don't know if I'll ever see them again,' you know and that that breaks my heart you know that's really tough. And so those kinds of things are really challenging'* ***Family carer 6, non-resident daughter, (baseline)***
**Theme 2: Reduced support:** <br>*'but you know, it's just um when, when will I be able to get a break, that's what I don't know'* ***Family carer 10, resident spouse, female (baseline)*** <br>*'definitely* (day care) *being open …because that at least you know I'd go round and see mum in any case but then I'd take her down to* [day care] *and then in 3 hours' time she would you know had company, something to eat um and just you know, somebody else seeing her, that 3 hours to me on that Thursday and a Tuesday meant a lot'* ***Family carer 7, non-resident, daughter (baseline)*** <br>'(peer support group) *have never reopened. It was on Zoom but X* (person with dementia) *couldn't partake in Zoom, he found that really difficult…. So, to try and understand and relate to lots of people in boxes, he just sat there and then he would look at me and say "how long have I got to sit here?" I said well you haven't got too, he couldn't understand that or partake in it so we stopped that so he's had nothing really. The Zooms stopped'* ***Family carer 10, resident spouse young onset, female (follow-up)*** <br>*'we've got friends and they would help out so the wife would come and uh look after X* (person with dementia) *and I go out with, with the husband. Um that stopped of course because um we can't even have someone come round to the house'* ***Family carer 3, resident spouse, male (follow up)***
**Theme 3: Deteriorating cognitive & physical health:** <br>*'the lack of stimulation I'm certain has had an impact on her ability to um join things up. Certainly her memory has got worse. … Um she just doesn't know what day it is um sometimes she doesn't know if it's morning or afternoon. And that's certainly has gotten worse since we've gone on lockdown'* ***Family carer 3, resident spouse, male (baseline)*** <br>*'she's sitting down a lot more, not getting out and about and moving so, so she's got more aches and pains'* ***Family carer 7, non-resident, daughter (baseline)***   *'not having the sorta will do doing things sorta thing, he's sorta getting not actually lazy but just getting the exhausted sorta doing something sorta, his routine is just laying down or sitting down sorta thing but not things sorta moving sorta doing anything'* ***Family carer 8, non-resident son, (baseline)*** <br>*'no, no it was, he was in decline before that'* ***Family carer 16, care home spouse, female***

For peer review only

Theme 4: Decreased carer wellbeing:

‘also during that time her need for help with personal care has increased, so I’m more and more tied down and there’s no easy way out of that… I have less and less time because I’m less and less able to leave X (person with dementia) *for any length of time, either to go out for a quick walk or even to do things in the house’* ***Family carer 3, resident spouse, male (follow-up)***

‘with the pandemic, obviously mum’s carer, my helpers who used to call round of an evening obviously most of them then couldn’t go round so it was myself going round practically every evening’ ***Family carer 7, non-resident, daughter (baseline)***

‘I’ve been very weepy when we were in lockdown, I’ve not been coping nearly as well as normal because it’s been so hard because of having no time for me, and no break. I think I’m just exhausted, exhausted and no time to myself’ ***Family carer 10, resident spouse young onset, female (baseline)***

‘you know it’s, he just kinda like suffocates me all the time. And I just get, get really frustrated. And I just, sometimes I just don’t wanna be here, I wanna be anywhere else but here’ ***Family carer 2, resident spouse, female (baseline)***

Theme 5: Difficulties understanding Covid-19:

‘at that time, he was just really really unhappy because he didn’t understand what was going on and why he couldn’t do things and why our sons couldn’t come round and yeah, I mean why he couldn’t see our little grandson, why he couldn’t get his haircut etc, … his lack of understanding of why he can’t go and get his haircut and why he can’t go to a shop and buy something that he wants. You know, when I say we can’t do that he says “Why? Why on earth not?” You know he can’t understand that and explain, I explain time and time again but often he doesn’t get it’ ***Family carer*** **10, resident spouse young onset, female (follow up)**

‘and therefore she doesn't quite understand all of it. And uh just keeps repeating and repeating that you know 'Why can't we?” ***Family carer 4, resident spouse, male (baseline)***

Theme 6: Limited impact for some:

‘He doesn’t, he doesn’t, he’s got no anxiety so he doesn’t worry about people not being around the house coz he doesn’t remember’ ***Family carer 11, resident, daughter (baseline)***

‘she’s just basically quite unaware of what’s happening’ ***Family carer 3, resident spouse, male (follow-up)***

Theme 7: Trust & relationship with care home:

‘and she's being looked after and it’s the best place for her so therefore I suppose it, it, it does me help you know, it makes me feel better knowing that she's, she's okay you know… the homes very good, they put me straight onto X (person with dementia) *and they let me know how she's getting on’* ***Carer 13, care home spouse, male***

‘and they’ve never, I asked, I asked every week, “can I see X (person with dementia)*, can I see X* (person with dementia)*” “oh we don’t know where the* (I-Pad) *tablet is, it must be in the manager's office”. Hey ho they have lost it, it's now, I keep asking for this tablet and no one knows where it is’* ***Carer 15, care home spouse, female***

For peer review only

Ethics statement

Approval for this study was given by NHS Health Research Authority London Queen Square Research Ethics Committee (15/LO/0046).

Contributorship statement

Conception and design: SD, SB, NT, Data collection: NA, JP, BF, EA, GT, Analysis: SD, JP, GT, Manuscript drafting: SD, JP, SD, BF, SB, NF, YF, NT. All authors read and approved the final manuscript.

Competing interests statement

The authors have no competing interests

Funding

This study was funded by the National Institute for Health Research (NIHR) Applied Research Collaboration Kent, Surrey, Sussex (grant number: N/A). Funding was also provided by Sussex Partnership NHS Foundation Trust and Brighton & Sussex Medical School

The views expressed are those of the authors and not necessarily those of the NHS, the NIHR, University of Sussex, University of Brighton or the Department of Health and Social Care

Data-sharing statement

Data sharing is available through contacting the corresponding author

Acknowledgements:

We would like to thank the contribution of the members of the lived experience advisory group; Julia Fountain, Ellen Jones, Claire Parker, Fiona McGhee, Tina and Ian Wakeford

For peer review only

We would also like to thank the following staff for their contribution to the study; Dr Sam Robertson, Rachel Russell, Natalie Portwine, Tamsin Eperson, Alice Russell, Jacob Reichental, Anomita Karim, Marcela Carvajal

References:

1. Prince M, Knapp M, Guerchet M, McCrone P, Prina M, Comas-Herrera A, et al. Dementia UK London: Alzheimer's Society 2014.
2. Greenberg NE, Wallick A, Brown LM. Impact of COVID-19 pandemic restrictions on community-dwelling caregivers and persons with dementia. Psychological Trauma: Theory, Research, Practice, and Policy. 2020;12(S1):S220.
3. Alzheimer's Society. Alzheimer's Society launches Emergency Appeal to support vulnerable people affected by dementia London 2020 [Available from: https://www.alzheimers.org.uk/news/2020-04-02/alzheimers-society-launches-emergency-appeal-support-vulnerable-people-dementia.
4. Alzheimer's Society. Social Contact Briefing 2020 [Available from: https://www.alzheimers.org.uk/sites/default/files/2020-06/Social-Contact-Briefing_June-2020.pdf.
5. 3 Nations Dementia Working Group. The Impact of COVID-19 on People Affected by Dementia Webinar Series 2020 [cited 2021 24th January 2021]. Available from: https://www.3ndementiawg.org/all-about-our-webinars/.
6. Giebel C, Cannon J, Hanna K, Butchard S, Eley R, Gaughan A, et al. Impact of COVID-19 related social support service closures on people with dementia and unpaid carers: a qualitative study. Aging & Mental Health. 2020:1-8.
7. TIDE, Law 4 Dementia Carers. Impact of the Coronavirus Act 2020 and legal issues on Carers of People with Dementia 2020 [Available from: https://www.tide.uk.net/wp-content/uploads/2020/10/L4DC-Covid-19-Report.pdf.
8. Saxena S, Orley J. Quality of life assessment: The world health organization perspective. European Psychiatry. 1997;12:263s-6s.
9. Banerjee S, Farina N, Daley S, Grosvenor W, Hughes L, Hebditch M, et al. How do we enhance undergraduate healthcare education in dementia? A review of the role of innovative approaches and development of the Time for Dementia Programme. International journal of geriatric psychiatry. 2017;32(1):68-75.
10. Smith SC, Murray J, Banerjee S, Foley B, Cook JC, Lamping DL, et al. What constitutes health-related quality of life in dementia? Development of a conceptual framework for people with dementia and their carers. International Journal of Geriatric Psychiatry: A journal of the psychiatry of late life and allied sciences. 2005;20(9):889-95.
11. Vellone E, Piras G, Venturini G, Alvaro R, Cohen MZ. Quality of life for caregivers of people with Alzheimer's disease living in Sardinia, Italy. Journal of Transcultural Nursing. 2012;23(1):46-55.
12. Jones C, Edwards RT, Hounsome B. Qualitative exploration of the suitability of capability based instruments to measure quality of life in family carers of people with dementia. ISRN family medicine. 2014;2014:919613-.
13. Daley S, Murray J, Farina N, Page TE, Brown A, Basset T, et al. Understanding the quality of life of family carers of people with dementia: Development of a new conceptual framework. International journal of geriatric psychiatry. 2019;34(1):79-86.
14. Sommerlad A SS, Singh-Manoux A,, Lewis G LGP, https://doi.org/10.1371/ Me, journal.pmed.1002862. Association of social contact with dementia and cognition: 28-year follow-up of the Whitehall II cohort study. PLoS Med. 2019;16(8).
15. Vellone E, Piras G, Talucci C, Cohen MZ. Quality of life for caregivers of people with Alzheimer's disease. Journal of Advanced Nursing. 2008;61(2):222-31.

For peer review only

16. Bruce DG, Paley GA, Nichols P, Roberts D, Underwood PJ, Schaper F. Physical disability contributes to caregiver stress in dementia caregivers. The Journals of Gerontology Series A: Biological Sciences and Medical Sciences. 2005;60(3):345-9.
17. Shanley C, Russell C, Middleton H, Simpson-Young V. Living through end-stage dementia: The experiences and expressed needs of family carers. Dementia. 2011;10(3):325-40.
18. Lynn-sMcHale DJ, Deatrick JA. Trust Between Family and Health Care Provider. Journal of Family Nursing. 2000;6(3):210-30.
19. Sloane PD, Zimmerman S, Williams CS, Reed PS, Gill KS, Preisser JS. Evaluating the quality of life of long-term care residents with dementia. The Gerontologist. 2005;45(suppl 1):37-49.

For peer review only

Figure 1: Relationship between themes

For peer review only

For peer review only

Figure 1: Relationship between themes

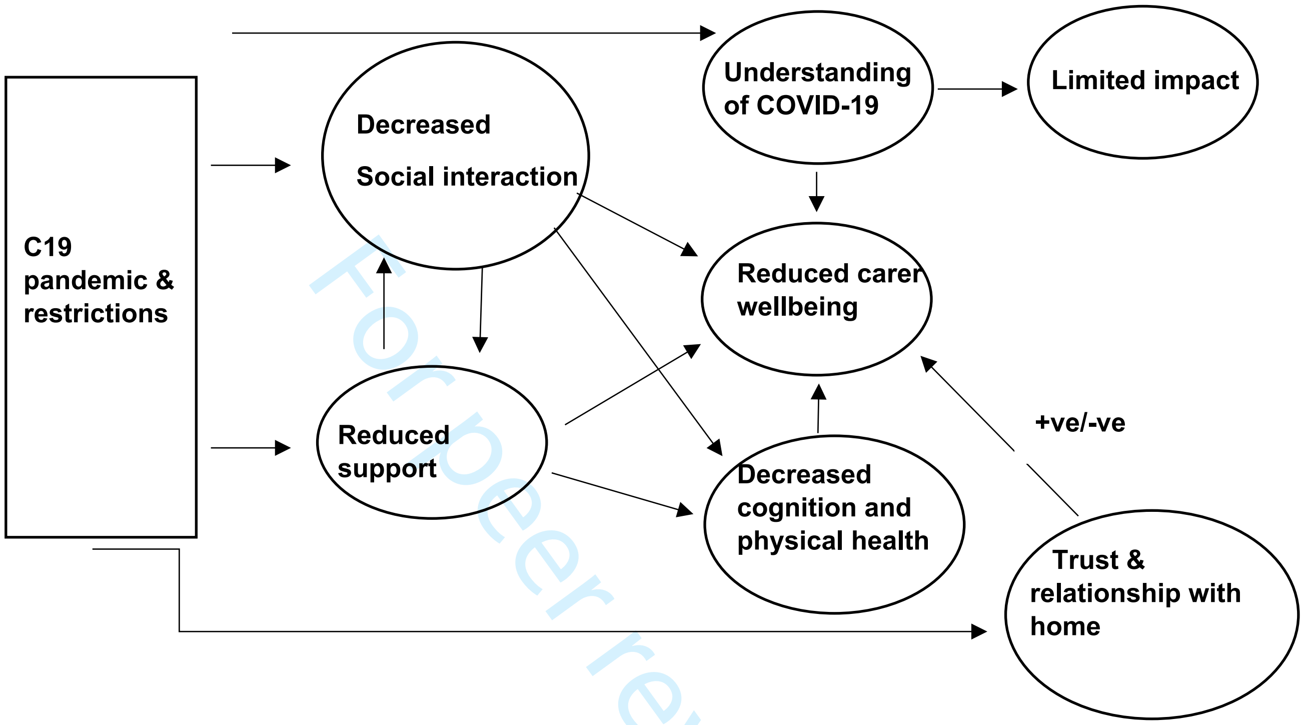

For peer review only

Standards for Reporting Qualitative Research (SRQR)*

http://www.equator-network.org/reporting-guidelines/srqr/

Title and abstract

	Page/line no(s).
**Title** - Concise description of the nature and topic of the study Identifying the study as qualitative or indicating the approach (e.g., ethnography, grounded theory) or data collection methods (e.g., interview, focus group) is recommended	1
**Abstract** - Summary of key elements of the study using the abstract format of the intended publication; typically includes background, purpose, methods, results, and conclusions	2

Introduction

**Problem formulation** - Description and significance of the problem/phenomenon studied; review of relevant theory and empirical work; problem statement	4
**Purpose or research question** - Purpose of the study and specific objectives or questions	5

Methods

**Qualitative approach and research paradigm** - Qualitative approach (e.g., ethnography, grounded theory, case study, phenomenology, narrative research) and guiding theory if appropriate; identifying the research paradigm (e.g., postpositivist, constructivist/ interpretivist) is also recommended; rationale**	6
**Researcher characteristics and reflexivity** - Researchers' characteristics that may influence the research, including personal attributes, qualifications/experience, relationship with participants, assumptions, and/or presuppositions; potential or actual interaction between researchers' characteristics and the research questions, approach, methods, results, and/or transferability	7
**Context** - Setting/site and salient contextual factors; rationale**	6
**Sampling strategy** - How and why research participants, documents, or events were selected; criteria for deciding when no further sampling was necessary (e.g., sampling saturation); rationale**	6
**Ethical issues pertaining to human subjects** - Documentation of approval by an appropriate ethics review board and participant consent, or explanation for lack thereof; other confidentiality and data security issues	6
**Data collection methods** - Types of data collected; details of data collection procedures including (as appropriate) start and stop dates of data collection and analysis, iterative process, triangulation of sources/methods, and modification of procedures in response to evolving study findings; rationale**	6

For peer review only - http://bmjopen.bmj.com/site/about/guidelines.xhtml

**Data collection instruments and technologies** - Description of instruments (e.g., interview guides, questionnaires) and devices (e.g., audio recorders) used for data collection; if/how the instrument(s) changed over the course of the study	6
**Units of study** - Number and relevant characteristics of participants, documents, or events included in the study; level of participation (could be reported in results)	7
**Data processing** - Methods for processing data prior to and during analysis, including transcription, data entry, data management and security, verification of data integrity, data coding, and anonymization/de-identification of excerpts	6
**Data analysis** - Process by which inferences, themes, etc., were identified and developed, including the researchers involved in data analysis; usually references a specific paradigm or approach; rationale**	6
**Techniques to enhance trustworthiness** - Techniques to enhance trustworthiness and credibility of data analysis (e.g., member checking, audit trail, triangulation); rationale**	

Results/findings

**Synthesis and interpretation** - Main findings (e.g., interpretations, inferences, and themes); might include development of a theory or model, or integration with prior research or theory	7-11
**Links to empirical data** - Evidence (e.g., quotes, field notes, text excerpts, photographs) to substantiate analytic findings	11-12

Discussion

**Integration with prior work, implications, transferability, and contribution(s) to the field -** Short summary of main findings; explanation of how findings and conclusions connect to, support, elaborate on, or challenge conclusions of earlier scholarship; discussion of scope of application/generalizability; identification of unique contribution(s) to scholarship in a discipline or field	12-13
**Limitations** - Trustworthiness and limitations of findings	13

Other

**Conflicts of interest** - Potential sources of influence or perceived influence on study conduct and conclusions; how these were managed	2
**Funding** - Sources of funding and other support; role of funders in data collection, interpretation, and reporting	2

*The authors created the SRQR by searching the literature to identify guidelines, reporting standards, and critical appraisal criteria for qualitative research; reviewing the reference lists of retrieved sources; and contacting experts to gain feedback. The SRQR aims to improve the transparency of all aspects of qualitative research by providing clear standards for reporting qualitative research.

For peer review only

For peer review only - http://bmjopen.bmj.com/site/about/guidelines.xhtml

**The rationale should briefly discuss the justification for choosing that theory, approach, method, or technique rather than other options available, the assumptions and limitations implicit in those choices, and how those choices influence study conclusions and transferability. As appropriate, the rationale for several items might be discussed together.

Reference:

O'Brien BC, Harris IB, Beckman TJ, Reed DA, Cook DA. **Standards for reporting qualitative research: a synthesis of recommendations.** *Academic Medicine*, Vol. 89, No. 9 / Sept 2014 DOI: 10.1097/ACM.0000000000000388

For peer review only

For peer review only - http://bmjopen.bmj.com/site/about/guidelines.xhtml

BMJ Open

What factors have influenced quality of life in people with dementia and their family carers during the COVID-19 pandemic: a qualitative study

Journal:	*BMJ Open*
Manuscript ID	bmjopen-2021-053563.R1
Article Type:	Original research
Date Submitted by the Author:	29-Nov-2021
Complete List of Authors:	Daley, Stephanie; Brighton and Sussex Medical School, Centre for Dementia Studies<br>Akarsu, Nazire; Sussex Partnership NHS Foundation Trust<br>Armsby , Elise; Sussex Partnership NHS Foundation Trust<br>Farina, Nicolas; Brighton and Sussex Medical School, Centre for Dementia Studies<br>Feeney, Yvonne; Brighton and Sussex Medical School, Centre for Dementia Studies<br>Fine , Bethany; Sussex Partnership NHS Foundation Trust<br>Hughes, Laura; Brighton and Sussex Medical School<br>Pooley, Joanna; Sussex Partnership NHS Foundation Trust<br>Tabet, Naji; Brighton and Sussex Medical School<br>Towson , Georgia; Sussex Partnership NHS Foundation Trust<br>Banerjee, Sube; Brighton and Sussex Medical School, Centre for Dementia Studies
**Primary Subject Heading**:	Geriatric medicine
Secondary Subject Heading:	Mental health, Qualitative research
Keywords:	Dementia < NEUROLOGY, Old age psychiatry < PSYCHIATRY, COVID-19

SCHOLARONE™ Manuscripts

I, the Submitting Author has the right to grant and does grant on behalf of all authors of the Work (as defined in the below author licence), an exclusive licence and/or a non-exclusive licence for contributions from authors who are: i) UK Crown employees; ii) where BMJ has agreed a CC-BY licence shall apply, and/or iii) in accordance with the terms applicable for US Federal Government officers or employees acting as part of their official duties; on a worldwide, perpetual, irrevocable, royalty-free basis to BMJ Publishing Group Ltd ("BMJ") its licensees and where the relevant Journal is co-owned by BMJ to the co-owners of the Journal, to publish the Work in this journal and any other BMJ products and to exploit all rights, as set out in our licence.

The Submitting Author accepts and understands that any supply made under these terms is made by BMJ to the Submitting Author unless you are acting as an employee on behalf of your employer or a postgraduate student of an affiliated institution which is paying any applicable article publishing charge ("APC") for Open Access articles. Where the Submitting Author wishes to make the Work available on an Open Access basis (and intends to pay the relevant APC), the terms of reuse of such Open Access shall be governed by a Creative Commons licence – details of these licences and which Creative Commons licence will apply to this Work are set out in our licence referred to above.

Other than as permitted in any relevant BMJ Author's Self Archiving Policies, I confirm this Work has not been accepted for publication elsewhere, is not being considered for publication elsewhere and does not duplicate material already published. I confirm all authors consent to publication of this Work and authorise the granting of this licence.

For peer review only

What factors have influenced quality of life in people with dementia and their family carers during the COVID-19 pandemic: a qualitative study

Authors:

Dr Stephanie Daley[a]

Nazire Akarsu[a]

Elise Armsby[b]

Dr Nicolas Farina[a]

Yvonne Feeney[a]

Bethany Fine[b]

Dr Laura Hughes[a]

Joanna Pooley[a]

Dr Naji Tabet[a]

Georgia Towson[a]

Professor Sube Banerjee[c]

Affiliations and addresses:

a. Centre for Dementia Studies, Brighton and Sussex Medical School, UK
b. Dementia Research Unit, Sussex Partnership NHS Foundation Trust, UK
c. Faculty of Health, University of Plymouth, UK

Corresponding Author

Dr Stephanie Daley

Address: Brighton & Sussex Medical School, Centre for Dementia Studies, Trafford Building, Room 101, University of Sussex, Falmer, BN1 9RY**.**

Email: s.daley@bsms.ac.uk

Tel: 00 447393 761947

For peer review only

Abstract

Objectives: The COVID-19 pandemic has led to significant disruption to health and social care services. For people with dementia and their family carers this is problematic, as a group who rely upon timely and responsive services to live well with the condition. This study has sought to understand how COVID-19 has affected the quality of life of people diagnosed with dementia and their family carers

Design: Our mixed-methods study was nested in a larger cohort study of an education programme, Time for Dementia

Setting: The study took place in the South-East of England.

Participants: Existing study participants, family carers were approached about the COVID-19 nested study. A purposeful sample of participants were invited to take part in in-depth qualitative interview. The sample included family carers in a range of different caring situations.

Measurement: Interviews were undertaken remotely by telephone. Interviews sought to understand quality of life before the pandemic, impact of the restrictions on both the person with dementia and family carer, role of services and other agencies as well as supportive factors. Data were analysed using thematic analysis.

Results: 16 family carers were interviewed. Seven themes were identified from our analysis 1) decreased social interaction, 2) reduced support, 3) deteriorating cognitive & physical health for the person with dementia, 4) decreased carer well-being 5) difficulties understanding COVID-19 restrictions, 6) limited impact for some, and 7) trust and relationship with care home. There was little change between themes during the first and second wave of national lockdowns.

Conclusions: Our study provides an understanding the short-term impact of COVID-19 on the quality of life of people with dementia and their family carers. Our findings suggest that recovery between the first and second wave of the restrictions did not automatically take place.

For peer review only

Strengths and limitations of this study

- Strengths of this study are the inclusion of a range of family carers across different caring situations, as well as data collection in 'real time'.
- A further strength is that the study did not have an *a priori* hypothesis, and as such took an inductive 'bottom up' approach to data collection and analysis, with strong lived experience involvement.
- Limitations include the use of proxy reports from family carers about the impact of the pandemic on the QoL of people with dementia, rather than self-report
- The study sample lacks ethnic diversity as well as those with a recent diagnosis of dementia.

For peer review only

Introduction

The COVID-19 pandemic and healthcare policy has been hugely impactful upon people with dementia. Dementia has been shown to contribute towards a higher risk of mortality from COVID-19 (1-3), and healthcare policy towards those with dementia, particularly to those living in care homes has been shown to contribute towards increased mortality, distress and poor care delivery (4, 5). Additionally, regional, and national lockdowns have led to significant disruption to health and social care services, which in some area still remain. For people with dementia and their family carers this is problematic. There are currently 850,000 people with dementia living in the UK (6) who rely upon timely and responsive services to live well with the condition. The abrupt closure and reduction of many health and community support services arising from the pandemic is of concern for this vulnerable group. Many governments introduced a range of restrictions (shielding for the most medically vulnerable, self-isolation, and social distancing) to help reduce the spread of COVID-19, and to protect older and vulnerable people. Commentaries exist about how the these restrictions may be affecting people with dementia and carers (7) however the full and longer term impact of these restrictions has yet to be assessed.

Emerging research highlights that along with the disruption to formal dementia services, restrictions have led to a further loss of support from family and friends for people with dementia and family carers (8). Since the first national lockdown in March 2020 in the UK, there has been an increased number of people telephoning the support services provided by the Alzheimer's Society due to concerns about increased cognitive and functional deterioration in family members with dementia (9). Additionally, the the UK advocacy group; 3 Nations Dementia Working Group) reported that that people with dementia attending their regular webinars expressed significant concern about the withdrawal of services leading to a sense of abandonment, along with anxiety about the potential for a permanent loss of services (10). This theme of abandonment was also identified in a review of over 5000 Twitter tweets by people with dementia and their carers during the pandemic, due in part to the enforced isolation and lack of contact with others, particularly for those in care homes (11).

Empirical data is emerging. . One qualitative study which looked at the impact of reduced social support with 42 carers and 8 people with dementia identified that it was the 'suddenness' of the reduction in services that had significantly affected both family carers and people with dementia (12). This research identified three key themes: loss of control, uncertainty (about current and future provision), and a need to adapt to 'the new normal' (including remote rather than face to face contact). A survey of 191 family carers of people

For peer review only

with dementia found an 83% increase of caring tasks as a consequence of COVID-19, with over 50% reporting that they felt unable to fulfil the caring role adequately (13). Increased carer stress, fatigue and burnout has also been found in other studies (3, 14, 15). In one study 21 family carers, severe loneliness, and an overwhelming sense of loss and concern about worsening cognition in the person with dementia was identified (16). Concern about cognition and the impact of restrictions upon cognition and neuropsychiatric symptoms has been identified more widely (17, 18). For example, one spanish study identified an increase in neuropsychiatric symptoms in people with Alzheimer's Disease and Mild-Cognitive impairment during 5 weeks of lockdown, with agitation, apathy and aberrant motor behaviour being most prominent (19).

The emerging literature highlights the substantial impact of the loss of both formal and voluntary sector services, a fear of services not resuming, increased carer burden, and concern about increased cognitive decline in the person with dementia. We have been keen to understand the specific impact of the pandemic on Quality of Life (QoL) given that living with a diagnosis of dementia, or caring for somebody with dementia is highly impactful upon all of the factors known to affect QoL, such as physical health, psychological state, independence, social relationships, personal beliefs and environmental supports (20). Whilst factors influencing QoL in both people with dementia and their carers differ, their outcomes are inextricably linked; with carers being a vital determinant of QoL and positive outcomes for people with dementia, and vice versa. A recent scoping review sought to assess the impact of the pandemic on Quality of Life (QoL) of people with dementia and their carers (21) through the assessment of both care provision and QoL data for both groups. This review identified three areas in which the pandemic had impacted on QoL; unmet and increasing care needs, increased stress and burden in carers of people with dementia, and finally a range of demographic factors, such as disease severity. This is perhaps not surprising, however we were keen to understand the impact more broadly and the relationship between factors. . Therefore, in this qualitative study we have aimed to understand how COVID-19 has affected the QoL of people diagnosed with dementia and their family carers

Methods

Study design

Our study was nested in a larger cohort study, Time for Dementia (22). Time for Dementia is an undergraduate education programme in the South-East of England, whereby people with

For peer review only

dementia and their carer teach healthcare students about living with the condition. During the pandemic period, there has been remote contact (video conferencing or telephone) between carers, the person with dementia and their family carer on a termly basis.

Sample and setting

Study participants lived in Kent, Surrey and Sussex. The region has the highest proportion of older people in the UK, with 50,000 people current living with dementia. The region is an area of extremes; it includes both rural areas that tend to be more affluent but more socially isolated, and urban areas that have higher levels of deprivation. Notably, the region includes coastal areas that feature in the highest decile of deprivation.

We recruited family carers of people with a diagnosis of dementia from the Time for Dementia study. The study definition of family carers included married and unmarried partners, children, siblings, extended family and close friends. The term 'family carers' is since it was preferred by our lived experience advisory group. We sampled for variation in characteristics of family carers, using the socio-demographic information provided by participants. From discussion with our lived experience advisory group, we agreed that these characteristics should include: resident and non-resident family carers, spousal and non-spousal family carers, those who were shielding, and family carers of people with dementia in care homes. Due to the difficulty in assessing capacity over the telephone, people with dementia were not interviewed as part of this study. The study was approved by the NHS Health Research Authority London Queen Square Research Ethics Committee (15/LO/0046).

Procedure

Participants in the Time for Dementia evaluation were approached by a research worker about the COVID-19 nested study. Those who were interested were sent a study information sheet, and verbal consent was obtained by those who wished to take part. Quantitative measures of QoL were undertaken, along with socio-demographic information and are reported separately. A purposeful sample of participants were asked about undertaking an in-depth qualitative interview. The sample included family carers in a range of different caring situations.

Topic guides were developed from an initial review of the literature, and key topic areas included: life before the pandemic, impact of the restrictions on both the person with dementia and family carer, role of services and other agencies and supportive factors (see

For peer review only

supplementary file). Additionally, the topic guide for interviews were amended to explore change over the pandemic period, and also for family carers of people living in a care home.

Interviews were carried out between May and November 2020 by JP, SD and NA. Twelve interviews were undertaken in the first lockdown (May and June 2020), and ten interviews were undertaken during the second lockdown (November 2020). Six participants were interviewed twice, and family carers of people living with dementia living in a care home (n=4) were interviewed once during the second lockdown. Interviews were stopped when data reached thematic saturation. Interviews were undertaken by telephone and lasted between 30 and 60 minute. All interviews were audio-recorded, transcribed verbatim and checked for accuracy.

Analysis

Data analysis commenced in July 2020 using thematic analysis. The process started with descriptive coding of two transcripts by two researchers (SD and JP) who manually coded the transcripts by giving descriptive codes to meaningful segments of text. The researchers met to review their respective preliminary codes and identify areas of differences and to develop an initial framework. The remaining 10 transcripts (from the first round of interviews) were coded to develop a focussed framework. The topic guide was amended at this stage, to gain further understanding of developing themes and to explore change over time. During the second phase of the analysis (second round of interviews), 6 transcripts were coded by SD and JP. GT and SD undertook a separate analysis of the care home transcripts, using the existing study coding framework, generating additional codes and themes as necessary.

The computer software package, NVivo 12 (QSR International, 2018) was used to enable the systematic collation and review of the data grouped within each code. The researchers met regularly to compare coding and data between existing and new transcripts in order to check on the use of codes for consistency and areas of uncertainty. The researchers also jointly reviewed data collated within each code, to identify relationships between codes, as well as areas of uncertainty, coming to an agreement once discussed. In the last phase of the analysis, the care home data was incorporated, and a final framework was produced from which seven over-arching themes were identified.

Rigour in the research process was supported in two ways. First, the lead researcher (SD) met frequently with the researchers for academic supervision to review coding, the development of themes and reflexivity. Second, all researchers kept fieldwork diaries.

For peer review only

Patient and public involvement

People with dementia and their family carers were involved throughout the research process though a specific Lived Experience Advisory Group (LEAP) which was established for the study. The group reviewed the study design, participant-facing documentation, the topic guide on two occasions as well as emerging and final themes. The group have also been involved in developing dissemination material for participants, as well as for local services and stakeholders.

Results

The 16 participants were predominantly White British/European (94%), spousal carers (64%). Half of participants were co-resident with the person with dementia (50%). Table

Table 1 shows the participant characteristics.

Insert Table 1 here

Themes

Our analysis identified seven themes: 1) decreased social interaction, 2) reduced support, 3) deteriorating cognitive & physical health, 4) decreased carer well-being 5) difficulties understanding COVID-19 restrictions, 6) limited impact for some, and 7) trust and relationship with care home. Themes are illustrated in more depth in Table 2.

Insert Table 2 here

These themes were all identified in relation to perceived impact of COVID-19 on the QoL of the person with dementia and family carer. For the majority of themes, there was little change between the first and second lockdown. With the exception of family carers of people with dementia living in care homes, those interviewed highlighted day-to-day life before the pandemic as including opportunities for social interaction and for support for both the person with dementia and family carer with benefits to each. Many of the themes identified are inter-related both between themselves and between the person with dementia and their carer, as shown in Figure 1.

Insert Figure 1 here

Theme 1: Decreased social interaction

All family carers reported that the significant reduction in face to face social interaction and contact with others impacted upon QoL for themselves and the person with dementia. Feelings of loneliness and isolation were frequently reported by family carers, especially for

For peer review only

those who would have ordinarily relied upon social contact as a source of respite from their caring role. Lack of social interaction led to sense of reduced informal support (from others) and was impactful across the majority of other themes identified in the analysis.

Family carers expressed a sense of time being short for the person with dementia, and precious time with family members being lost. Concern about the potential risks of catching the virus appeared to result in isolation for many family carers who reported that they had avoided social opportunities, declined support from services both for themselves and the person with dementia.

Family carers of those in care homes were all spousal carers, and all reported that being unable to see the person with dementia was very impactful on their QoL, leading to significant feelings of loneliness.

Theme 2: Reduced support

Family carers stated that pre-COVID informal face-to-face support provided by friends and family was highly valued, and positively impactful on their own QoL as well as the QoL of the person with dementia. Since the COVID-19 pandemic, this type of support had reduced for many, primarily due to social distancing regulations, including shielding. In the absence of face-to-face support, family carers recognised the benefit of remote contact with friends and family. An increased reliance on technology (such as phone and video calls) was seen as being problematic for both the family and for the person with dementia. Family carers reported that it is more difficult to offload fully about current caring challenges by phone or video calls, either because the person with dementia was present or because there was less available time. For those with dementia who were unfamiliar with technology such as video calls, family carers reported that using them was often confusing, and of limited value.

A sharp reduction of services such as day care, respite, community groups and non-urgent healthcare was widely reported, accompanied by concerns about the future resumption of support services. For a small number of those interviewed, formal respite services had continued or had resumed, and this was highly valued.

Family carers also reported that some professional support groups adapted to provide some services remotely, and whilst this was valued, there was dissatisfaction with the depth of connection and support possible through remote contact.

For peer review only

Due to COVID-19, the process of obtaining a care home place had become more difficult, with three family carers reporting that the transition into the care home had ultimately been made following an acute hospital admission.

Theme 3: Deteriorating cognitive and physical health

Many of the family carers interviewed expressed concern about increased cognitive decline in the person with dementia, linking this to a lack of stimulation, from reduced social interaction with others (family, friends and dementia support services). Several of carers interviewed noted that the decline in cognition could also have been attributable due to the natural progression of the condition.

Where the person with dementia was community dwelling, family carers reported that reduced levels of social and support activity disrupted the routine of the person with dementia, negatively impacting upon their motivation and fatigue levels.

Family carers reported worsening physical health in the person with dementia for those who were community dwelling. Reduced mobility was highlighted as a particular concern, linked to the wider lack of activity, along with an increased rate of joint pain, and for some, an increase in urinary tract infections.

Theme 4: Decreased carer well-being

For most family carers, the pandemic had led to increased caring tasks arising from increased dependence of the person with dementia and a reduced level (service or family/friend) support. Increasing caring, along with the lack of respite opportunities for a break had led to a sense of burnout for some. Feelings of frustration, anger and resentment from some of those interviewed was expressed, increasing over time between the first and second lockdown.

Additionally, for family carers, increased caring tasks and the need for direct supervision of the person with dementia impacted upon their ability to meet their own needs. The majority of family carers interviewed described a loss of freedom in being able to undertake the activities which they wanted to undertake, and a sense of a loss of control and certainty. Some of those interviewed described feeling suffocated, lonely and frustrated.

For peer review only

Theme 5: Difficulties understanding COVID-19 restrictions

Difficulties in understanding the pandemic and its related restrictions was reported as being problematic for people with dementia. Carers reported that for some of those with dementia, the restrictions were often forgotten, or that they were too confusing, or difficult to understand. For many family carers, they reported that they frequently needed to explain the pandemic and remind the person with dementia about restrictions (for example; need to avoid close contact with others) when outside the home. This also applied to people with dementia in care homes, as carers stated that they were constantly having to explain why they could not visit in person, or why they were not allowed to touch the person with dementia. This often led to the person with dementia becoming upset, or in some cases angry.

Theme 6: Limited impact from the pandemic

For a small number of people with dementia, carers reported that there was no perceived impact arising from the pandemic due their lack of understanding of the situation, and not remembering that they were not seeing family and friends less often.

Theme 7: Trust/relationship with care home

Where the person with dementia was in a care home, family carers identified the importance of maintaining a good relationship and trust with the care home as being pivotal to their QoL, due to the family carer being unable to visit the home. Carers stated that care homes who facilitated good communication through phone calls, video calls or emails enabled a greater sense of ease and confidence. Having confidence in the care provided, and knowing that the person with dementia was safe and well looked after was perceived as essential for family carer QoL. One participant described poor care being delivered to the person with dementia, along with poor communication as having a negative impact on their QoL.

Maintaining contact between the carer and the person with dementia was achieved in a variety of ways, including the use of garden ‘pods’, or telephone contact. However, one participant reported that no alternative means of maintaining contact with the person with dementia was provided. The ability to maintain contact with the person with dementia impacted carer QoL, and increased contact with the person with dementia was associated with enhanced satisfaction with care and improved QoL for carers.

For peer review only

Discussion

We have identified seven themes arising from the COVID-19 pandemic which family carers reported to have affected QoL for people with dementia and family carers.

Reduced social interaction was the most impactful factor influencing QoL, and whilst its identification as a factor is not surprising, its mediating role on other factors is of note. For people with dementia; the value of social relationships on QoL in terms of social support, social integration and companionship (23) is well known. For family carers, the positive impact of social interaction from family and social networks on QoL is also well-established (24-26). However, in terms of the impact of the COVID-19 pandemic, the effect appears to be two-pronged, firstly it is sudden yet on-going sense of *loss of* social contact with meaningful others which is impactful, as well as actual impact of reduced contact with others. For the person with dementia, the reduced social contact was seen by the majority of family carers as increasing cognitive impairment in the person with dementia, reinforcing the importance of social contact as a protective factor against cognitive decline (27). Reduced social interaction with others, and the perceived impact of this on the cognition of the person with dementia increased the burden on family carers, along with the loss of personal freedom and a decreased ability to meet their own needs and their overall well-being. Loss of personal freedom has been identified as a factor in the QoL of family members of people with dementia (24, 26, 28). Reduced family carer well-being is of concern, as if prolonged, this may impair the ability of family carers to look after the person with dementia, and may lead to an increased rate of transition into care homes. Our findings indicate that the resumption of face-to-face social support services for people with dementia and their family carers should be prioritised. Furthermore a lessening of restrictions will bring with it opportunities for families and friends to socialise, which of highly valued for this group The impact of reduced formal support on QoL is consistent with emerging research (3, 12, 29, 30), and fears about services not resuming (10). Finally, difficulty understanding local guidance for restrictions, and for the person with dementia, the rationale behind the restrictions was also identified in an analysis of Twitter tweets (11)

Our finding that the social restrictions from the pandemic have negatively impacted upon the physical health of the person with dementia has not yet been identified elsewhere, indicating that more research in this area is required. Interestingly, whilst worsening family carer physical health is factor known to influence the QoL of carers of people with dementia (31) this was not identified in this study, possibly because of the relatively short time period, or alternatively because it may have existed pre-COVID-19.

For peer review only

Within the context of a care home setting, the importance of trust and a good relationship between family carers and staff is clear. In our study, three of the four people with dementia in a care home had been transferred to the care home during the COVID-19 pandemic. Notwithstanding some of the wider challenges experienced by care homes in relation to COVID-19, the lack of face-to-face contact between family carers and staff can be seen as an additional obstacle to be overcome. The need for care homes to meaningfully involve family carers is care decisions is recognised as being significant in the development of both trust and a good relationship (32), and ordinarily trust in care staff evolves over time as a process (33), therefore, the absence of a ‘normal’ face-to-face relationship with family carers has been problematic. Further research into the experiences of care home staff and people with dementia living in care homes during the pandemic would be useful to understand these experiences more fully

Finally, whilst this study provides an understanding the short-term impact of COVID-19 on people with dementia and their family carers, we do not know what the longer-term impact might be. We also do not know how a return to ‘normal’ might be achieved, or what the ‘new normal’ might look like for people with dementia and their family carers, and what might help or hinder this process. It is also not clear from this study whether the impact of the pandemic is similar across different contexts, for example, with shorter or less restrictive periods of national lockdown or in different areas. It does appear that recovery from the impact of the first wave of restrictions did not automatically take place, and anecdotally we are aware that reductions in social support services has been prolonged, with many services still not having fully resumed. The provision of formal support during the pandemic to the study sample was highly variable. The increased use of technology and potential opportunities to deliver services to people with dementia and their carers during the pandemic is evident (34-36). However, the efficacy of these new models of delivery is not known, and there remain very few data about how dementia services have responded overall to the pandemic, and how they might adapt going forward to meet the post-pandemic challenges for this group.

These findings have a number of implications for services for people with dementia and their carers during period of national lockdowns or enforced social restrictions. First, there is a need for services to proactively to identify people with dementia and their carers who are struggling, and from this to provide more targeted support. Second, services need to ensure that people with dementia remain connected to other people, and provide support where necessary to do this. For some people, this may include setting up or adapting communication technology. Third, care homes should maintain regular communication with

For peer review only - http://bmjopen.bmj.com/site/about/guidelines.xhtml

carers, and should proactively support active connection between people with dementia and their carers. Fourth, services have a key role in developing and disseminating dementia friendly summaries of complex national or local guidance. Finally, services should encourage and enable people with dementia and their carers to keep physically active, thereby helping to maintain good physical health and mobility.

There are three strengths to this study. First, we were able to include a wide range of family carers in order to understand the impact of COVID-19 across a range of caring situations. Second, interviews were undertaken during both national lockdown periods with data being collected in 'real time', without reliance on recall. Third, the study did not have an *a priori* hypothesis, and as such took an inductive 'bottom up' approach to data collection and analysis, supported by strong lived experience involvement. This has helped to strengthen the validity of the findings and kept the study alert to the current concerns of those living with dementia during this unprecedented period.

There are four main limitations to this study. First, we have taken proxy reports from family carers about the impact of the pandemic on the QoL of people with dementia. QoL is a subjective appraisal and it is well known that proxy reports assess QoL differently to self-appraisal by people with dementia (37). It is therefore possible that people with dementia may have identified other factors as being more or less impactful during the pandemic. Proxy-reports for the people with dementia living in care homes are also less likely to be accurate, as contact between family carers and the person with dementia may have been too limited for a more informed assessment of QoL. Second, there is a lack of ethnic diversity in the study sample. The limits the generalizability of these findings to the wide group of Black, Asian and other ethnic minority group members who have been disproportionately negatively affected by the pandemic overall, and for whom the impact for those living with dementia may be more substantial. Third, this study did not include family carers of somebody of a recent diagnosis of dementia. All of those interviewed had been caring for the person with dementia for a period of time and therefore, the factors identified were additional to their on-going experience of living with dementia and the degree to the pandemic impacted upon this. This study therefore does not specifically aid understanding to the impact of COVID-19 on the QoL of those with a recent diagnosis of dementia. Fourth, we did not collect demographic information on household income and/or deprivation therefore limiting the generalisability of the study findings to different context and reducing potential observations about influencing economic and social factors.

For peer review only

However, this study does provide a new and valuable information that contributes usefully to the emerging evidence base on the impact of the COVID-19 restrictions on people with dementia and their family carers. The finding and associated recommendations can help commissioners and service providers in developing and delivering the robust post-pandemic support that is needed by this vulnerable group.

For peer review only

Table 1: Participant characteristics

Characteristic	Mean (SD) (Range)	n	%
**Age**	65.63 (9.88) (48 – 83)	16	
**Gender**			
Female		9	56.3
**Ethnicity**			
White British/European		15	93.8
Asian/ Asian British		1	6.3
**Marital status**			
Married		10	62.5
Cohabiting		2	12.5
Separated / Divorced		2	12.5
Never married/ Single		2	12.5
**Relationship to person with dementia**			
Spouse/partner		10	62.5
Son/daughter		6	37.5
**Currently employed**			
Yes		4	25.0
**Current/ last occupation**			
Managers/directors/officials		3	18.8
Professional		3	18.8
Associate professional/ technical		2	12.5
Administrative/ secretarial		5	31.3
Skilled trades		2	12.5
Caring/leisure/service		1	6.3
**Geographical location**			
Urban		1	6.3
Rural		7	43.8
Suburbs		8	50.0
**Person with dementia**			
**Type of dementia**			
Alzheimer's disease		4	25.0
Vascular dementia		3	18.8
Early onset Alzheimer's disease		2	12.5
Mixed		7	43.8
**Residence**			
Co-resident with carer		8	50.0
Non-resident		8	50.0
**Person with dementia**			
Community dwelling		12	75.0
Care home resident		4	25.0
**Time since diagnosis**	3.4 years (2.20) (1.1 - 7.5)		
Less than 1 year		0	0.0
Between 1 and 3 years		9	56.3
Between 3 and 5 years		2	12.5
Over 5 years		5	31.3

For peer review only

Table 2 Summary of themes

**Themes/Quotes**
**Theme 1: Decreased Social interaction:**<br><br>*'very very isolating really, both for me and mum really erm, coz she misses, she obviously misses seeing everyone'* ***Family carer 12, resident son, (baseline)***<br><br>*'um well I miss it, miss it terribly because I'm quite gregarious and I do miss it, probably y'know somebody, not having people to kind of talk to and have a laugh about things and um just generally just to offload really, um y'know that makes a difference'* ***Family carer 2, resident spouse, female (follow-up)***<br><br>*'the other main one really was has not being able to see his grandchildren. And because it's hard for anybody to know you know and I've sat with him while he's cried and gone "I don't know if I'll ever see them again,' you know and that that breaks my heart you know that's really tough. And so those kinds of things are really challenging'* ***Family carer 6, non-resident daughter, (baseline)***
**Theme 2: Reduced support:**<br><br>*'but you know, it's just um when, when will I be able to get a break, that's what I don't know'* ***Family carer 10, resident spouse, female (baseline)***<br><br>*'definitely* (day care) *being open …because that at least you know I'd go round and see mum in any case but then I'd take her down to* [day care] *and then in 3 hours' time she would you know had company, something to eat um and just you know, somebody else seeing her, that 3 hours to me on that Thursday and a Tuesday meant a lot'* ***Family carer 7, non-resident, daughter (baseline)***<br><br>'(peer support group) *have never reopened. It was on Zoom but X* (person with dementia) *couldn't partake in Zoom, he found that really difficult…. So, to try and understand and relate to lots of people in boxes, he just sat there and then he would look at me and say "how long have I got to sit here?" I said well you haven't got too, he couldn't understand that or partake in it so we stopped that so he's had nothing really. The Zooms stopped'* ***Family carer 10, resident spouse young onset, female (follow-up)***<br><br>*'we've got friends and they would help out so the wife would come and uh look after X* (person with dementia) *and I go out with, with the husband. Um that stopped of course because um we can't even have someone come round to the house'* ***Family carer 3, resident spouse, male (follow up)***
**Theme 3: Deteriorating cognitive & physical health:**<br><br>*'the lack of stimulation I'm certain has had an impact on her ability to um join things up. Certainly her memory has got worse. … Um she just doesn't know what day it is um sometimes she doesn't know if it's morning or afternoon. And that's certainly has gotten worse since we've gone on lockdown'* ***Family carer 3, resident spouse, male (baseline)***<br><br>*'she's sitting down a lot more, not getting out and about and moving so, so she's got more aches and pains'* ***Family carer 7, non-resident, daughter (baseline)***<br>*'not having the sorta will do doing things sorta thing, he's sorta getting not actually lazy but just getting the exhausted sorta doing something sorta, his routine is just laying down or sitting down sorta thing but not things sorta moving sorta doing anything'* ***Family carer 8, non-resident son, (baseline)***<br><br>*'no, no it was, he was in decline before that'* ***Family carer 16, care home spouse, female***

Theme 4: Decreased carer wellbeing:

'also during that time her need for help with personal care has increased, so I'm more and more tied down and there's no easy way out of that… I have less and less time because I'm less and less able to leave X (person with dementia) *for any length of time, either to go out for a quick walk or even to do things in the house'* ***Family carer 3, resident spouse, male (follow-up)***

'with the pandemic, obviously mum's carer, my helpers who used to call round of an evening obviously most of them then couldn't go round so it was myself going round practically every evening' ***Family carer 7, non-resident, daughter (baseline)***

'I've been very weepy when we were in lockdown, I've not been coping nearly as well as normal because it's been so hard because of having no time for me, and no break. I think I'm just exhausted, exhausted and no time to myself' ***Family carer 10, resident spouse young onset, female (baseline)***

'you know it's, he just kinda like suffocates me all the time. And I just get, get really frustrated. And I just, sometimes I just don't wanna be here, I wanna be anywhere else but here' ***Family carer 2, resident spouse, female (baseline)***

Theme 5: Difficulties understanding Covid-19:

'at that time, he was just really really unhappy because he didn't understand what was going on and why he couldn't do things and why our sons couldn't come round and yeah, I mean why he couldn't see our little grandson, why he couldn't get his haircut etc, … his lack of understanding of why he can't go and get his haircut and why he can't go to a shop and buy something that he wants. You know, when I say we can't do that he says "Why? Why on earth not?" You know he can't understand that and explain, I explain time and time again but often he doesn't get it' ***Family carer*** **10, resident spouse young onset, female (follow up)**

'and therefore she doesn't quite understand all of it. And uh just keeps repeating and repeating that you know 'Why can't we?" ***Family carer 4, resident spouse, male (baseline)***

Theme 6: Limited impact for some:

'He doesn't, he doesn't, he's got no anxiety so he doesn't worry about people not being around the house coz he doesn't remember' ***Family carer 11, resident, daughter (baseline)***

'she's just basically quite unaware of what's happening' ***Family carer 3, resident spouse, male (follow-up)***

Theme 7: Trust & relationship with care home:

'and she's being looked after and it's the best place for her so therefore I suppose it, it, it does me help you know, it makes me feel better knowing that she's, she's okay you know… the homes very good, they put me straight onto X (person with dementia) and they let me know how she's getting on' ***Carer 13, care home spouse, male***

'and they've never, I asked, I asked every week, "can I see X (person with dementia), can I see X (person with dementia)" "oh we don't know where the (I-Pad) *tablet is, it must be in the manager's office". Hey ho they have lost it, it's now, I keep asking for this tablet and no one knows where it is'* ***Carer 15, care home spouse, female***

For peer review only

Ethics statement

Approval for this study was given by NHS Health Research Authority London Queen Square Research Ethics Committee (15/LO/0046).

Contributorship statement

Conception and design: SD, SB, NT, Data collection: NA, JP, BF, EA, GT, Analysis: SD, JP, GT, Manuscript drafting: SD, JP, SD, BF, SB, NF, YF, NT. All authors read and approved the final manuscript.

Competing interests statement

The authors have no competing interests

Funding

This study was funded by the National Institute for Health Research (NIHR) Applied Research Collaboration Kent, Surrey, Sussex (grant number: N/A). Funding was also provided by Sussex Partnership NHS Foundation Trust and Brighton & Sussex Medical School

The views expressed are those of the authors and not necessarily those of the NHS, the NIHR, University of Sussex, University of Brighton or the Department of Health and Social Care

Data-sharing statement

Data sharing is available through contacting the corresponding author

Acknowledgements:

We would like to thank the contribution of the members of the lived experience advisory group; Julia Fountain, Ellen Jones, Claire Parker, Fiona McGhee, Tina and Ian Wakeford

For peer review only

We would also like to thank the following staff for their contribution to the study; Dr Sam Robertson, Rachel Russell, Natalie Portwine, Tamsin Eperson, Alice Russell, Jacob Reichental, Anomita Karim, Marcela Carvajal

References:

1. Hariyanto TI, Putri C, Situmeang RFV, Kurniawan A. Dementia is a predictor for mortality outcome from coronavirus disease 2019 (COVID-19) infection. European Archives of Psychiatry and Clinical Neuroscience. 2021;271(2):393-5.
2. Williamson EJ, Walker AJ, Bhaskaran K, Bacon S, Bates C, Morton CE, et al. Factors associated with COVID-19-related death using OpenSAFELY. Nature. 2020;584(7821):430-6.
3. Bacsu J-DR, O'Connell ME, Webster C, Poole L, Wighton MB, Sivananthan S. A scoping review of COVID-19 experiences of people living with dementia. Canadian Journal of Public Health. 2021;112(3):400-11.
4. Estabrooks CA, Straus SE, Flood CM, Keefe J, Armstrong P, Donner GJ, et al. Restoring trust: COVID-19 and the future of long-term care in Canada. FACETS. 2020;5(1):651-91.
5. Psychiatrists RCo. Impact of the COVID-19 pandemic on hospital care for people with dementia: Feedback from carers/families and hospital leads for dementia,. London: Royal College of Psychiatrists 2020
6. Prince M, Knapp M, Guerchet M, McCrone P, Prina M, Comas-Herrera A, et al. Dementia UK London: Alzheimer's Society 2014.
7. Greenberg NE, Wallick A, Brown LM. Impact of COVID-19 pandemic restrictions on community-dwelling caregivers and persons with dementia. Psychological Trauma: Theory, Research, Practice, and Policy. 2020;12(S1):S220.
8. Alzheimer's Society. Alzheimer's Society launches Emergency Appeal to support vulnerable people affected by dementia London 2020 [Available from: https://www.alzheimers.org.uk/news/2020-04-02/alzheimers-society-launches-emergency-appeal-support-vulnerable-people-dementia.
9. Alzheimer's Society. Social Contact Briefing 2020 [Available from: https://www.alzheimers.org.uk/sites/default/files/2020-06/Social-Contact-Briefing_June-2020.pdf.
10. 3 Nations Dementia Working Group. The Impact of COVID-19 on People Affected by Dementia Webinar Series 2020 [cited 2021 24th January 2021]. Available from: https://www.3ndementiawg.org/all-about-our-webinars/.
11. Bacsu J-D, O'Connell ME, Cammer A, Azizi M, Grewal K, Poole L, et al. Using twitter to understand the COVID-19 experiences of people with Dementia: infodemiology study. Journal of Medical Internet Research. 2021;23(2):e26254.
12. Giebel C, Cannon J, Hanna K, Butchard S, Eley R, Gaughan A, et al. Impact of COVID-19 related social support service closures on people with dementia and unpaid carers: a qualitative study. Aging & Mental Health. 2020:1-8.
13. TIDE, Law 4 Dementia Carers. Impact of the Coronavirus Act 2020 and legal issues on Carers of People with Dementia 2020 [Available from: https://www.tide.uk.net/wp-content/uploads/2020/10/L4DC-Covid-19-Report.pdf.
14. Cohen G, Russo MJ, Campos JA, Allegri RF. Living with dementia: increased level of caregiver stress in times of COVID-19. International Psychogeriatrics. 2020;32(11):1377-81.
15. Savla J, Roberto KA, Blieszner R, McCann BR, Hoyt E, Knight AL. Dementia caregiving during the "stay-at-home" phase of COVID-19 pandemic. The Journals of Gerontology: Series B. 2021;76(4):e241-e5.
16. Harris ML, Titler MG. Experiences of Family Caregivers of People with Dementia during the COVID-19 Pandemic. Western Journal of Nursing Research.0(0):01939459211055773.

17. Palermo G, Tommasini L, Baldacci F, Del Prete E, Siciliano G, Ceravolo R. Impact of coronavirus disease 20 19 pandemic on cognition in Parkinson's disease. Movement Disorders. 2020;35(10):1717-8.
18. Roach P, Zwiers A, Cox E, Fischer K, Charlton A, Josephson CB, et al. Understanding the impact of the COVID-19 pandemic on well-being and virtual care for people living with dementia and care partners living in the community. Dementia. 2021;20(6):2007-23.
19. Lara B, Carnes A, Dakterzada F, Benitez I, Piñol-Ripoll G. Neuropsychiatric symptoms and quality of life in Spanish patients with Alzheimer’s disease during the COVID-19 lockdown. European journal of neurology. 2020;27(9):1744-7.
20. Saxena S, Orley J. Quality of life assessment: The world health organization perspective. European Psychiatry. 1997;12:263s-6s.
21. Masterson-Algar P, Allen MC, Hyde M, Keating N, Windle G. Exploring the impact of Covid-19 on the care and quality of life of people with dementia and their carers: A scoping review. Dementia.0(0):14713012211053971.
22. Banerjee S, Farina N, Daley S, Grosvenor W, Hughes L, Hebditch M, et al. How do we enhance undergraduate healthcare education in dementia? A review of the role of innovative approaches and development of the Time for Dementia Programme. International journal of geriatric psychiatry. 2017;32(1):68-75.
23. Smith SC, Murray J, Banerjee S, Foley B, Cook JC, Lamping DL, et al. What constitutes health-related quality of life in dementia? Development of a conceptual framework for people with dementia and their carers. International Journal of Geriatric Psychiatry: A journal of the psychiatry of late life and allied sciences. 2005;20(9):889-95.
24. Vellone E, Piras G, Venturini G, Alvaro R, Cohen MZ. Quality of life for caregivers of people with Alzheimer’s disease living in Sardinia, Italy. Journal of Transcultural Nursing. 2012;23(1):46-55.
25. Jones C, Edwards RT, Hounsome B. Qualitative exploration of the suitability of capability based instruments to measure quality of life in family carers of people with dementia. ISRN family medicine. 2014;2014:919613-.
26. Daley S, Murray J, Farina N, Page TE, Brown A, Basset T, et al. Understanding the quality of life of family carers of people with dementia: Development of a new conceptual framework. International journal of geriatric psychiatry. 2019;34(1):79-86.
27. Sommerlad A SS, Singh-Manoux A,, Lewis G LGP, https://doi.org/10.1371/ Me, journal.pmed.1002862. Association of social

contact with dementia and cognition: 28-year

follow-up of the Whitehall II cohort study. PLoS Med. 2019;16(8).
28. Vellone E, Piras G, Talucci C, Cohen MZ. Quality of life for caregivers of people with Alzheimer's disease. Journal of Advanced Nursing. 2008;61(2):222-31.
29. Cagnin A, Di Lorenzo R, Marra C, Bonanni L, Cupidi C, Laganà V, et al. Behavioral and psychological effects of coronavirus disease-19 quarantine in patients with dementia. Frontiers in psychiatry. 2020;11:916.
30. Dening KH, Lloyd-Williams M. Minimising long-term effect of COVID-19 in dementia care. The Lancet. 2020;396(10256):957-8.
31. Bruce DG, Paley GA, Nichols P, Roberts D, Underwood PJ, Schaper F. Physical disability contributes to caregiver stress in dementia caregivers. The Journals of Gerontology Series A: Biological Sciences and Medical Sciences. 2005;60(3):345-9.
32. Shanley C, Russell C, Middleton H, Simpson-Young V. Living through end-stage dementia: The experiences and expressed needs of family carers. Dementia. 2011;10(3):325-40.
33. Lynn-sMcHale DJ, Deatrick JA. Trust Between Family and Health Care Provider. Journal of Family Nursing. 2000;6(3):210-30.

For peer review only

34. Di Lorito C, Bosco A, Goldberg SE, Nair R, O'Brien R, Howe L, et al. Protocol for the process evaluation of the Promoting Activity, Independence and Stability in Early Dementia (PrAISED), following changes required by the COVID-19 pandemic. BMJ open. 2020;10(8):e039305.
35. Goodman-Casanova JM, Dura-Perez E, Guzman-Parra J, Cuesta-Vargas A, Mayoral-Cleries F. Telehealth home support during COVID-19 confinement for community-dwelling older adults with mild cognitive impairment or mild dementia: survey study. Journal of medical Internet research. 2020;22(5):e19434.
36. Lai FH-y, Yan EW-h, Yu KK-y, Tsui W-S, Chan DT-h, Yee BK. The protective impact of telemedicine on persons with dementia and their caregivers during the COVID-19 pandemic. The American Journal of Geriatric Psychiatry. 2020;28(11):1175.
37. Sloane PD, Zimmerman S, Williams CS, Reed PS, Gill KS, Preisser JS. Evaluating the quality of life of long-term care residents with dementia. The Gerontologist. 2005;45(suppl 1):37-49.

For peer review only

Figure 1: Relationship between themes

For peer review only

For peer review only

Figure 1: Relationship between themes

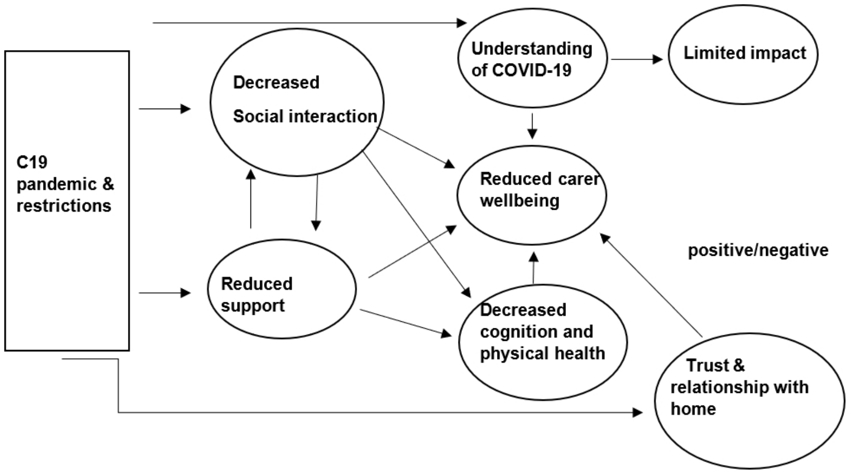

Figure 1: Relationship between themes

513x313mm (57 x 57 DPI)

TfD C19 Study

<u>Topic Guide/Interview schedule</u>

Interview by phone or video conferencing

Introduction

My name is …... I am a researcher on the COVID-19 study

Remind participant about study – to understand the impact of C19 on the quality of life of people living with dementia.

Aim for today:

- *to complete an interview / have an informal chat about your experiences of COVID-19*

If you are happy to go ahead

- *is it convenient to do it today?*
- *Audio recording*
- *Anonymized and confidential*
- *40-50 minutes*
- *Keep to time*
- *Can stop / take a break at any time*

Do you have any questions?

Are you happy to go ahead?

To do: Test dictaphone

Record carer ID, date, start time

Introduction

I am going to start by asking you a question about lift before the pandemic and then ask you to tell me more about the general impact of the C19 restrictions on you and the person you care for (use their name/relationship) . I will then ask you about their (person with dementia) experiences in more depth and then finish the interview with exploring your experiences in more detail.

For peer review only - http://bmjopen.bmj.com/site/about/guidelines.xhtml

1) Can you tell me a little bit about the life before the pandemic for you and for the person who you care for (name)

2) Can you tell me impact of the C19 restrictions on your day to day life?

3) Can you tell me more about the general impact of C19 restrictions on day to day life on the person (name) who you care for?

4) In more detail, what has been the impact on the (name) person with dementia?

- *(Prompts) Positive/Negative? What has been most impactful?*
- *What has the person with dementia understood about what has been happening? Especially if there has been no/reduced contact with carer?*
- *What has been the impact of reduced social interaction with others? What about the loss of physical contact*
- *Has there been any impact on their dementia? If so, in what way? Why is this?*
- *What has been the impact on their routine? What has been the impact on their physical health (including mobility)?*
- *Has there been any impact on their general well-being and mental health – how would you say that they are feeling?*
- *How they found any support or solutions which have been particularly helpful/useful?*

5) Impact of the carer

- *What has been the impact on you? (Positive/Negative)*

- *Prompts (to explore) What impact has there been on:*
- *Your ability to meet your own personal needs*
- *Your well-being,*
- *Your relationship with the person with dementia – (If you are not living with/seeing the person with dementia – how has this been for you? What might be the impact on your relationship?)*
- *Your feelings about the future*
- *how supported do you feel?*
- *What impact is there on your social networks/isolation?*
- *How has this impacted upon your sense of control?*
- *How has the impact on the person with dementia affected you?*
- *Has the person with dementia received a DNR (do not resuscitate) notification – if yes, what is the impact of this on you?*

- *Any other areas which they feel have been impacted upon?*

TfD C19 study carer topic guide

For peer review only

For peer review only - http://bmjopen.bmj.com/site/about/guidelines.xhtml

- *How have you found any support or solutions (national local) which have been particularly helpful/useful?*

6) Other?

Anything which we haven't discussed which might be relevant?

Is there anything from the pandemic experience which you would like to 'keep'

7) Debrief

- *How did you find the interview/how are you feeling now?*
- *Would you be interested in doing another interview later in the year should we be able to do some further follow?*
- *Re-iterate that he/she can call researcher if he/she feels distressed after call*
- *Re-iterate that we will do a follow up call if he/she has been distressed*
- *What is he/she going to do next?*

For peer review only

For peer review only - http://bmjopen.bmj.com/site/about/guidelines.xhtml

Standards for Reporting Qualitative Research (SRQR)*

http://www.equator-network.org/reporting-guidelines/srqr/

Title and abstract

	Page/line no(s).
**Title** - Concise description of the nature and topic of the study Identifying the study as qualitative or indicating the approach (e.g., ethnography, grounded theory) or data collection methods (e.g., interview, focus group) is recommended	1
**Abstract** - Summary of key elements of the study using the abstract format of the intended publication; typically includes background, purpose, methods, results, and conclusions	2

Introduction

**Problem formulation** - Description and significance of the problem/phenomenon studied; review of relevant theory and empirical work; problem statement	4
**Purpose or research question** - Purpose of the study and specific objectives or questions	5

Methods

**Qualitative approach and research paradigm** - Qualitative approach (e.g., ethnography, grounded theory, case study, phenomenology, narrative research) and guiding theory if appropriate; identifying the research paradigm (e.g., postpositivist, constructivist/ interpretivist) is also recommended; rationale**	6
**Researcher characteristics and reflexivity** - Researchers' characteristics that may influence the research, including personal attributes, qualifications/experience, relationship with participants, assumptions, and/or presuppositions; potential or actual interaction between researchers' characteristics and the research questions, approach, methods, results, and/or transferability	7
**Context** - Setting/site and salient contextual factors; rationale**	6
**Sampling strategy** - How and why research participants, documents, or events were selected; criteria for deciding when no further sampling was necessary (e.g., sampling saturation); rationale**	6
**Ethical issues pertaining to human subjects** - Documentation of approval by an appropriate ethics review board and participant consent, or explanation for lack thereof; other confidentiality and data security issues	6
**Data collection methods** - Types of data collected; details of data collection procedures including (as appropriate) start and stop dates of data collection and analysis, iterative process, triangulation of sources/methods, and modification of procedures in response to evolving study findings; rationale**	6

For peer review only

For peer review only - http://bmjopen.bmj.com/site/about/guidelines.xhtml

**Data collection instruments and technologies** - Description of instruments (e.g., interview guides, questionnaires) and devices (e.g., audio recorders) used for data collection; if/how the instrument(s) changed over the course of the study	6
**Units of study** - Number and relevant characteristics of participants, documents, or events included in the study; level of participation (could be reported in results)	7
**Data processing** - Methods for processing data prior to and during analysis, including transcription, data entry, data management and security, verification of data integrity, data coding, and anonymization/de-identification of excerpts	6
**Data analysis** - Process by which inferences, themes, etc., were identified and developed, including the researchers involved in data analysis; usually references a specific paradigm or approach; rationale**	6
**Techniques to enhance trustworthiness** - Techniques to enhance trustworthiness and credibility of data analysis (e.g., member checking, audit trail, triangulation); rationale**	

Results/findings

**Synthesis and interpretation** - Main findings (e.g., interpretations, inferences, and themes); might include development of a theory or model, or integration with prior research or theory	7-11
**Links to empirical data** - Evidence (e.g., quotes, field notes, text excerpts, photographs) to substantiate analytic findings	11-12

Discussion

**Integration with prior work, implications, transferability, and contribution(s) to the field -** Short summary of main findings; explanation of how findings and conclusions connect to, support, elaborate on, or challenge conclusions of earlier scholarship; discussion of scope of application/generalizability; identification of unique contribution(s) to scholarship in a discipline or field	12-13
**Limitations** - Trustworthiness and limitations of findings	13

Other

**Conflicts of interest** - Potential sources of influence or perceived influence on study conduct and conclusions; how these were managed	2
**Funding** - Sources of funding and other support; role of funders in data collection, interpretation, and reporting	2

*The authors created the SRQR by searching the literature to identify guidelines, reporting standards, and critical appraisal criteria for qualitative research; reviewing the reference lists of retrieved sources; and contacting experts to gain feedback. The SRQR aims to improve the transparency of all aspects of qualitative research by providing clear standards for reporting qualitative research.

For peer review only - http://bmjopen.bmj.com/site/about/guidelines.xhtml

**The rationale should briefly discuss the justification for choosing that theory, approach, method, or technique rather than other options available, the assumptions and limitations implicit in those choices, and how those choices influence study conclusions and transferability. As appropriate, the rationale for several items might be discussed together.

Reference:

O'Brien BC, Harris IB, Beckman TJ, Reed DA, Cook DA. **Standards for reporting qualitative research: a synthesis of recommendations.** *Academic Medicine*, Vol. 89, No. 9 / Sept 2014 DOI: 10.1097/ACM.0000000000000388

For peer review only

For peer review only - http://bmjopen.bmj.com/site/about/guidelines.xhtml
